# Assessment of Serum Endothelial Cell-Specific Molecule-1 (ESM-1, Endocan) Levels in Patients with Diabetic Retinopathy

**DOI:** 10.5152/eurasianjmed.2025.251038

**Published:** 2025-11-17

**Authors:** Mustafa Yıldırım, Emine Çinici, Muhammet Çelik, Selcan İçtaş

**Affiliations:** 1 Department of Ophthalmology, Atatürk University Faculty of Medicine, Erzurum, Türkiye; 2 Department of Biochemistry, Atatürk University Faculty of Medicine, Erzurum, Türkiye

**Keywords:** Diabetes mellitus, endocan, retinopathy

## Abstract

**Background:**

The study aims to evaluate serum endocan (endothelial cell-specific molecule-1) levels in patients with type 2 diabetes mellitus and to determine its relationship with disease activity by comparing it with a control group.

**Methods:**

Venous blood samples were collected from 63 patients with diabetic retinopathy and healthy individuals who visited the policlinic between January 1, 2020, and March 1, 2020. Serum endocan and HbA1c levels were evaluated in the patients.

**Results:**

The mean age of the patient and control groups was 62 ± 10.19 and 60.27 ± 5.53, respectively. No statistically significant difference was observed between the patient and control groups in terms of age and gender. Serum endocan levels were found to be statistically significantly higher in the patient group with diabetic retinopathy (*P* < .05).

**Conclusion:**

It was hypothesized that serum endocan levels could serve as a guiding parameter in assessing the severity of diabetic retinopathy.

**Main Points**

- Retinopathy is a severe microvascular complication of diabetes that can lead to blindness.
- Diabetic retinopathy, which occurs due to the disruption of the vascular endothelial structure, has 2 stages: proliferative and nonproliferative.
- Endocan may be an inflammatory marker indicating endothelial dysfunction.

## Introduction

Retinopathy is a severe microvascular complication of diabetes that can lead to blindness, with its incidence ranging from 2.2% to 12.7%.^1^ Diabetic retinopathy (DRP) has 2 primary stages: nonproliferative diabetic retinopathy (NPDR) and proliferative diabetic retinopathy (PDR). Nonproliferative diabetic retinopathy represents the initial stage of diabetic retinopathy and typically presents without symptoms. At this stage, the patient shows signs of pericyte loss, increased vascular permeability, and disruption of the inner blood-retinal barrier. Proliferative diabetic retinopathy is a severe stage in which neovascularization occurs, and changes that can progress to vitreous hemorrhages, fibrovascular membranes, and retinal detachment are observed.

The American Diabetes Association has defined diabetic retinopathy as a tissue-specific neurovascular complication seen in both types of diabetes.^2^ Hyperglycemia increases the production of glycation end products, reactive oxygen species, and proinflammatory cytokines (such as Tumor Necrosis Factor Alpha (TNF-α) and interleukin-6 (IL-6)) in plasma. These changes lead to dysfunction of the retinal neurovascular unit, characterized by microvasculopathy, pericyte cell loss, blood-retinal barrier dysfunction, inflammation, and neovascularization in the retina.^3^ Studies investigating the molecular mechanisms underlying vascular dysfunction in DRP, especially endothelial dysfunction, have shown that local ischemia in the retina increases the production of proinflammatory cytokines (interferon-γ, interleukin (IL)-8, IL-6, TNF-α, and IL-1β, etc. in the vitreous), growth factors (Vascular endothelial growth factor (VEGF), Platelet-derived growth factor (PDGF) etc.), adhesion molecules (Intercellular Adhesion Molecule-1 (ICAM-1) and vascular cellular adhesion molecules-1), chemokines (monocyte chemotactic protein-1), and receptors (CD40 and toll-like receptors) were shown to be released from inflammatory cells, retinal vascular cells, and glial cells.

Endothelial cell-specific molecule-1 (ESM-1, Endocan) is a soluble proteoglycan synthesized by vascular endothelial cells, particularly inflamed endothelium.^4^ It is involved in regulating the adhesion, migration, differentiation, and proliferation of various healthy and diseased cell types. Increased plasma ESM-1 values are found in inflammatory diseases and tumor activation. Therefore, plasma ESM-1 values may be a marker of endothelial cell dysfunction or activation. Endothelial cell-specific molecule-1 may serve as an inflammatory marker indicative of endothelial dysfunction.^5^ Diabetes causes endothelial damage and retinopathy through hyperglycemia and resulting inflammation. This article aims to demonstrate the effect of ESM-1, as measured in the serum of diabetic patients, on the development of retinopathy.

## Materials and Methods

The patient and control group members included in the study consisted of individuals examined in the ophthalmology outpatient clinic. The control group was selected from individuals who were examined in the ophthalmology outpatient clinic, were between the ages of 50 and 75, and did not have any systemic disease. There were 21 patients in the control group, 10 women and 11 men. The patient group was formed as a total of 63 type 2 diabetic patients, 21 with normal retina, 21 with NPDR, and 21 with PDR. Individuals with systemic diseases other than diabetes mellitus (DM) were not included in the study. The patient group included 28 women and 35 men, aged 50 to 75. The study received ethical approval from the Ethics Committee of Atatürk University Faculty of Medicine. (Approval no: B.30.2.ATA.0.01.00/162, Date: 26.03.2020). Written informed consent for publication of this article was obtained from 63 patients and 21 healthy individuals.

Histories were taken from these patients who came to the ophthalmology clinic with complaints of vision loss. The patients’ visual acuity, eye pressure (pneumotonometer), biomicroscopy, and panfundoscopy (Volk, USA), and anterior segment and fundus examinations were performed in detail by the same physician.

Optical coherence tomography (OCT-OPTUVOUE) and fundus fluorescein angiography (FFA-KOVA WX-10α) were used to distinguish proliferative and nonproliferative patients. Multiple microaneurysms, preretinal hemorrhages, hard-soft exudates, macular edema, and venous changes were evaluated as findings in favor of non-proDRP, while optic disc neovascularization, retinal neovascularization, vitreous hemorrhage, tractional retinal detachment, and iris-anterior chamber neovascularization were evaluated in favor of proDRP. A 4 mL blood sample was collected via an intravenous catheter before the FFA. Blood samples were transferred to tubes containing biochemistry and Potassium Ethylenediaminetetraacetic Acid (K-EDTA) gel. Samples were collected in the hospital’s Biochemistry Laboratory. Hemoglobin A1C was studied from blood samples containing K-EDTA. Serum samples obtained by centrifuging biochemistry tubes containing gel were stored at −80°C until analyzed. Serum ESM-1 levels were quantified using an Enzyme-Linked Immunosorbent Assay (ELISA) kit (BT LAB, Catalog no. E3160Hu, China).

### Statistical Analyses

Statistical analyses were evaluated using the SPSS 20.0 (SPSS, Chicago, IL, USA) program. The Kolmogorov–Smirnov test was employed to assess the normality of the parameters. *t*-test (independent test, Student’s *t*-test) and 1-way analysis of variance were used to compare serum ESM-1 values of patients and controls. The results are shown as mean ± SD. Statistical significance level between groups was accepted as *P* < .05.

## Results

The patient and control groups were assessed for demographic characteristics, and no statistically significant differences were observed (*P* > .05). Demographic data are shown in Table 1.

The best corrected visual acuity of all individuals in the control group was 10/10, and the intraocular pressure was within normal range (8-22 mmHg, assessed by pachymetry). Additionally, 5 out of 21 volunteers had posterior chamber intraocular lenses (IOLs) because they had undergone cataract surgery. Fundus examination showed normal findings in all participants in the control group.

In the diabetic group without retinopathy, visual acuity ranged from a minimum of 6/10 to a maximum of 10/10 on ophthalmological examination. Intraocular pressure was measured as normal in all patients. In 1 eye of 8 patients, it was determined that they had a posterior chamber IOL that had undergone cataract surgery before. In 1 eye of 3 patients, nuclear opacity was initially observed in the lens. In fundus examination, the retina was evaluated as normal.

In the ophthalmological examination of the diabetic group with retinopathy, the visual acuity ranged from a minimum of 2 mps (counting fingers per meter) to a maximum of 10/10. One patient was using antiglaucomatous medication due to high intraocular pressure, while the intraocular pressures of the other patients were normal. In 1 eye of 15 patients, it was observed that they had a posterior chamber IOL that had undergone cataract surgery before. In 5 patients, nuclear and posterior subcapsular opacities were observed in their unilateral lenses.

When serum ESM-1 levels were compared between the control group and the patient group, a statistically significant difference was observed in patients with retinopathy, whereas no significant difference was found in patients without retinopathy. The results are shown in Table 2. When HbA1c levels were compared between the patient and control groups, statistically significant differences were observed in all patient groups. The results are shown in Table 3. When patients with retinopathy were compared, no statistically significant difference was observed between patients with NPDR and PDR (*P* = .291).

**Figure 1.**
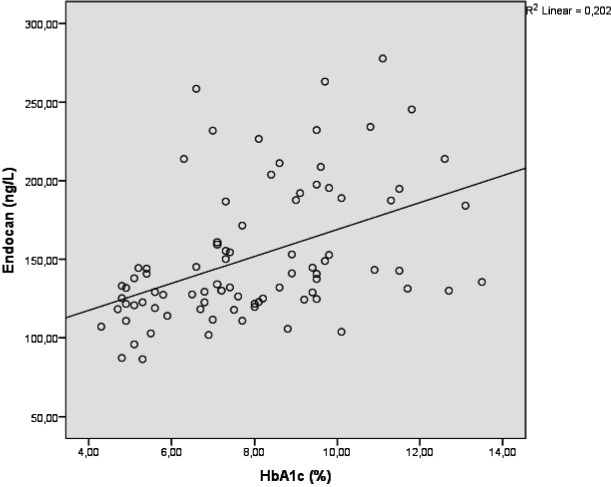
Correlation graph of ESM-1 and HbA1c levels.

A statistically significant correlation was found between ESM-1 and HbA1c levels, as shown in Figure 1 (*r* = 0.450, *P* < .001).

## Discussion

Diabetes is a chronic metabolic disease with significant life-threatening complications. Due to impaired metabolic control, advanced glycation end products caused by hyperglycemia accumulate in tissues, leading to intracellular signaling and gene expression changes and the release of proinflammatory molecules and free radicals that contribute to the pathology of microvascular complications of diabetes. This chronic low-grade inflammation in DM is a serious risk factor for micro- and macrovascular complications of DM. Even if diabetes does not develop, long-term hyperglycemia is known to increase plasma values of cytokines such as TNF-α, IL-6, and VEGF, causing retinopathy-like changes.

Retinopathy is a microvascular complication of diabetes that occurs through vascular endothelial dysfunction. It has been shown that diabetics have increased numbers of leukocytes, especially neutrophils, in their peripheral blood compared to their peers.^6^ Vascular endothelium plays a significant role in receptor/ligand interactions, inflammation caused by the secretion of different mediators, coagulation, angiogenesis, and tumor invasion. Endothelial cell-specific molecule-1 is a soluble chondroitin/dermatan sulfate proteoglycan known to function in angiogenesis, encoded by the *ESM-1* gene located on the long arm of the chromosome 4. It is considered an active mediator in the development of vascular disorders, inflammation, and endothelial dysfunction. VEGF is an effective mediator in angiogenesis due to its ability to stimulate endothelial cell proliferation, survival, and vascular permeability. Endothelial cell-specific molecule-1 facilitates endothelial cell activation through its role in augmenting VEGF-A signaling via Vascular Endothelial Growth Factor Receptor VEGFR-2 and increases endothelial permeability. It also activates components necessary for the adhesion and recruitment of leukocytes along activated endothelial cells. ICAM-1 mediates Lymphocyte Function-Associated Antigen-1 (LFA-1) LFA-1 binding to endothelial cells. This binding influences the suppression of cell-cell interactions, the attraction of circulating mononuclear cells to inflammatory regions, and adhesion and migration processes dependent on LFA-1. The ICAM-1/LFA-1 interaction further contributes to the regulation of cytotoxic lymphocyte and natural killer (NK) cell migration. Endothelial cell-specific molecule-1 binds to LFA-1 (CD11a/CD18) and regulates its association with ICAM-1, thereby inhibiting NK cell migration.

Among the studies investigating circulating ESM-1 levels in individuals with diabetes, Arman et al’s^7^ study evaluated ESM-1 values in 42 pre-diabetic patients and 42 healthy controls, finding that ESM-1 values were significantly reduced in the pre-diabetic patients. However, Klisic et al^8^ showed that there were comparable ESM-1 values between pre-diabetic patients and controls and that ESM-1 values were higher in patients with type 2 DM compared to both pre-diabetic patients and controls. In their study comparing ESM-1 levels among DM patients without retinopathy, patients with NPDR, patients with PDR, and healthy controls, Bozkurt et al^9^ evaluated serum ESM-1 values in diabetic patients with those in non-diabetic individuals and reported that ESM-1 levels were significantly higher in the diabetic group. It was also emphasized that it was higher in PDR than in NPDR. It has also been suggested that diabetic macular edema, a major cause of visual impairment apart from retinopathy, is linked to elevated blood ESM-1 levels.

Çelik et al^10^ reported that the values of ESM-1 in both blood and aqueous humor were higher in the diabetes and cataract groups, as well as in the diabetic retinopathy and cataract groups, compared to the normal group.

Asrar et al^11^ investigated the relationship between ESM-1 values in the vitreous of patients with PDR and disease activity and found that patients with proliferative diabetic retinopathy had higher ESM-1 levels than those without diabetes.

Shalwala et al^12^ presented that patients with proliferative DRP and nonproliferative DRP had similar ESM-1 values in vitreous samples and were significantly higher than those of patients without diabetes alone. No significant difference was observed in serum ESM-1 levels between the groups. Other studies have also reported that patients with type 2 DM had higher amounts of ESM-1 in the circulation than those without diabetes.^13^^,^^14^

In this study, similar to the study by Arman et al,^7^ it was found that the serum ESM-1 levels of diabetic patients were significantly higher than those of the control group. It was observed that ESM-1 levels increased as the retinopathy progressed, especially in diabetic patients. This suggests that serum ESM-1 levels may increase in accordance with the Diabetic Retinopathy (DR) stage and that ESM-1 may serve as a marker for DR progression. Additionally, this study found a positive correlation between DR stage and HbA1c levels. Many studies have indicated that high HbA1c levels are the strongest risk factor for DR progression.^15^^,^^16^ Glycemic control has been shown to have a significant impact on the presence and severity of DR.^17^ Andreasson et al^18^ showed a relationship was shown between HbA1c levels and DR stage, and it was stated that keeping HbA1c levels close to normal prolongs the development of DR. The current study supports these studies, and it was concluded that HbA1c levels, an indicator of impaired glycemic regulation, increase with the DR stage and are very significant in the progression of DR.

The main limitation of this study was its relatively small sample size. Although the sample size was relatively small, the number of patients in each group was sufficient to achieve statistical significance in the data analysis.

In conclusion, serum ESM-1 levels were found to be significantly higher in patients with diabetic retinopathy, and a statistically significant correlation was observed between ESM-1 and HbA1c levels. In summary, these findings suggest that ESM-1 may serve as an immunoinflammatory biomarker in diabetes and a warning for the development of potential complications.

## Figures and Tables

**Table 1.** Demographic Features of Patient and Control Groups

Parameter	Patient	Control	*P*
Age	62 ± 10.19	60.27 ± 5.53	.312
Gender (M/F)	35/28	11/10	.873
Systemic disease	DM	Null	

DM, diabetes mellitus; F, female; M, male.

**Table 2.** Comparison of Endothelial Cell-Specific Molecule-1 Serum Levels in Control Group and Diabetes Mellitus Patients

Participants (n)	ESM-1 (ng/mL)	*P*
Control (21) DM (21)	119.95 ± 17.02 137.96 ± 25.08	.367
Control (21) NPDR (21)	119.95 ± 17.02 160.15 ± 44.89	.003
Control (21) PDR (21)	119.95 ± 17.02 187.79 ± 46.73	.0001

DM, diabetes mellitus; ESM-1, endothelial cell-specific molecule-1; NPDR, nonproliferative diabetic retinopathy; PDR, proliferative diabetic retinopathy.

**Table 3.** Comparison of HbA1c Levels in Control Group and Diabetes Mellitus Patients

Participants (n)	HbA1c (%)	*P*
Control (21) DM (21)	5.16 ± 0.39 8.13 ± 1.61	.0001
Control (21) NPDR (21)	5.16 ± 0.39 8.20 ± 1.14	.0001
Control (21) PDR (21)	5.16 ± 0.39 10.35 ± 1.75	.0001

DM, diabetes mellitus; NPDR, nonproliferative diabetic retinopathy; PDR, proliferative diabetic retinopathy.

## Data Availability

The data presented in this study can be provided upon request from the corresponding author.

## References

[1] Thomas RL Halim S Gurudas S Sivaprasad S Owens DR. IDF diabetes atlas: a review of studies utilising retinal photography on the global prevalence of diabetes related retinopathy between 2015 and 2018. Diabetes Res Clin Pract. 2019;157:107840. (doi: 10.1016/j.diabres.2019.107840) 31733978

[2] Flaxel CJ Adelman RA Bailey ST Diabetic retinopathy preferred practice pattern®. Ophthalmology. 2020;127(1):P66 P145. (doi: 10.1016/j.ophtha.2019.09.025) 31757498

[3] Wang W Lo ACY. Diabetic retinopathy: pathophysiology and treatments. Int J Mol Sci. 2018;19(6):1816. (doi: 10.3390/ijms19061816) PMC6032159 29925789

[4] Sarrazin S Adam E Lyon M Endocan or endothelial cell specific molecule-1 (ESM-1): a potential novel endothelial cell marker and a new target for cancer therapy. Biochim Biophys Acta. 2006;1765(1):25 37. (doi: 10.1016/j.bbcan.2005.08.004) 16168566

[5] Balta S Mikhailidis DP Demirkol S Ozturk C Celik T Iyisoy A. Endocan: a novel inflammatory indicator in cardiovascular disease? Atherosclerosis. 2015;243(1):339 343. (doi: 10.1016/j.atherosclerosis.2015.09.030) 26448266

[6] Vozarova B Weyer C Lindsay RS Pratley RE Bogardus C Tataranni PA. High white blood cell count is associated with a worsening of insulin sensitivity and predicts the development of type 2 diabetes. Diabetes. 2002;51(2):455 461. (doi: 10.2337/diabetes.51.2.455) 11812755

[7] Arman Y Atici A Altun O Can the serum Endocan Level be used as a biomarker to predict subclinical athero- sclerosis in patients with Prediabetes? Arq Bras Cardiol. 2022;119(4):544 550. (doi: 10.36660/abc.20210797) 35946756 PMC9563878

[8] Klisic A Kavaric N Stanisic V Endocan and a novel score for dyslipidemia, oxidative stress and inflammation (DOI score) are independently correlated with glycated hemoglobin (HbA1c) in patients with prediabetes and type 2 diabetes. Arch Med Sci. 2020;16(1):42 50. 32051704 10.5114/aoms.2019.87541 PMC6963142

[9] Bozkurt E Gumus A Koban Y. Can serum endocan level predict stage of diabetic retinopathy? Retina-Vitreus. 2020;29(4):318 323. (doi: 10.37845/ret.vit.2020.29.58)

[10] Celik F Aydin S. Blood and aqueous humor phoenixin, endocan and spexin in patients with diabetes mellitus and cataract with and without diabetic retinopathy. Peptides. 2022;150:170728. (doi: 10.1016/j.peptides.2021.170728) 34971675

[11] Abu El-Asrar AM Nawaz MI De Hertogh G The angiogenic biomarker endocan is up regulated in proliferative diabetic retinopathy and correlates with vascular endothelial growth factor. Curr Eye Res. 2015;40(3):321 331. (doi: 10.3109/02713683.2014.921312) 24871583

[12] Shalwala A Lassalle P Caires NDF Elevation of vitreous endocan levels in proliferative diabetic retinopathy. Invest Ophthalmol Vis Sci. 2011;52:1295.

[13] Lv Y Zhang Y Shi W The association between endocan levels and subclinical atherosclerosis in patients with type 2 diabetes mellitus. Am J Med Sci. 2017;353(5):433 438. (doi: 10.1016/j.amjms.2017.02.004) 28502328

[14] Qiu CR Fu Q Sui J Analysis of serum endothelial cell-specific molecule 1 (endocan) level in type 2 diabetes mellitus with acute ST-segment elevation myocardial infarction and its correlation: a pilot study. Angiology. 2017;68(1):74 78. (doi: 10.1177/0003319716634581) 26927690

[15] Singh C Prasad SP Kaul S Motwani D Mishra A Padmakumar V. Association of HbA1c levels with diabetic retinopathy. Indian J Clin Exp Ophthalmol. 2021;7(2):339 345. (doi: 10.18231/j.ijceo.2021.067)

[16] Setareh J Hoseinzade G Khoundabi B Can the level of HbA1c predict diabetic retinopathy among type II diabetic patients? BMC Ophthalmol. 2022;22(1):415. (doi: 10.1186/s12886-022-02608-3) PMC9620629 36316667

[17] Kajal Seema S Jayalekshmi T Manasa S Prasenna M. Effect of glycemic control on diabetic retinopathy and diabetic macular edema: a prospective observational study. Int J Adv Med. 2021;8(2):177 182. (doi: 10.18203/2349-3933.ijam20210011)

[18] Andreasson R Ekelund C Landin-Olsson M Nilsson C. HbA1c levels in children with type 1 diabetes and correlation to diabetic retinopathy. J Pediatr Endocrinol Metab. 2018;31(4):369 374. (doi: 10.1515/jpem-2017-0417) 29494341

